# Soluble PD-L1: a potential dynamic predictive biomarker for immunotherapy in patients with proficient mismatch repair colorectal cancer

**DOI:** 10.1186/s12967-023-03879-0

**Published:** 2023-01-13

**Authors:** Yinjun He, Xiang Zhang, Ming Zhu, Wenguang He, Hanju Hua, Feng Ye, Xile Zhou, Nan Chen, Yandong Li, Weixiang Zhong, Guosheng Wu, Hui Cai, Weiqin Jiang

**Affiliations:** 1 Department of Colorectal Surgery, First Affiliated Hospital, Zhejiang University School of Medicine, Hangzhou, China; 2 College of Medicine, Zhejiang University, Hangzhou, China; 3 General Surgery Clinical Medical Center, Gansu Provincial Hospital, Lanzhou, China; 4 The First Clinical Medical College of Lanzhou University, Lanzhou, China; 5 Department of Radiology, First Affiliated Hospital, Zhejiang University School of Medicine, Hangzhou, China; 6 Department of Colorectal Surgery, Yuyao Hospital of Traditional Chinese Medicine, Ningbo, China; 7 Department of Pathology, First Affiliated Hospital, Zhejiang University School of Medicine, Hangzhou, China

**Keywords:** sPD-L1, Colorectal cancer, Biomarker, Combination immunotherapy

## Abstract

**Background:**

Circulating soluble programmed death ligand 1 (sPD-L1) can negatively regulate T-cell function and serve as a prognostic or predictive marker in a variety of cancers. However, rare studies have evaluated the potential roles of sPD-L1, and no study has estimated its predictive value for the efficacy of immune treatment in colorectal cancer (CRC).

**Methods:**

Plasma samples from 192 CRC patients were used to estimate correlations between clinicopathological features and sPD-L1, secreted PD-L1 (secPD-L1) and exosomal PD-L1 (exoPD-L1). Baseline and posttreatment sPD-L1 levels were also investigated in 55 patients with metastatic CRC (mCRC) treated with chemotherapy ± targeted therapy and 40 patients with proficient mismatch repair (pMMR) mCRC treated with combination immunotherapy. Both sPD-L1 and secPD-L1 were quantified by enzyme-linked immunosorbent assay, while exoPD-L1 was analyzed using flow cytometry.

**Results:**

secPD-L1 was the major component and positively correlated with sPD-L1 in CRC, while exoPD-L1 was almost undetectable. Higher levels of sPD-L1 were detected in patients with distant metastasis, especially those with distant lymph node metastasis and tissue combined positive score (CPS) instead of tumor proportion score (TPS). Chemotherapy or targeted therapy did not significantly impact sPD-L1 concentration. Progressive disease on combination immunotherapy was associated with an increase in sPD-L1 level, whereas no significant change was observed in patients with durable clinical benefit.

**Conclusion:**

sPD-L1 mainly consisted of secPD-L1, and its level was higher in patients with distant metastasis, especially distant lymph node metastasis and positive CPS. sPD-L1 is a potential dynamic marker to identify rapid progression on combination immunotherapy and avoid ineffective treatment for pMMR CRC.

**Supplementary Information:**

The online version contains supplementary material available at 10.1186/s12967-023-03879-0.

## Introduction

Growing evidence suggests that blocking the programmed cell death-1 (PD-1)/programmed cell death ligand-1 (PD-L1) pathway offers promising efficacy and prolonged survival in multiple types of tumors. While PD-1/PD-L1 monotherapy has revolutionized the treatment of deficient mismatch repair (dMMR) colorectal cancer (CRC) [1, 2], its combination with inhibitors of vascular endothelial growth factor receptor has reported a promising 15–33% response rate in proficient mismatch repair (pMMR) cohorts [3–5]. However, predictive biomarkers for immunotherapy are scarce.

PD-L1 can be expressed on the membranous surface of multiple cells, including tumor cells, immune cells and other cells in the tumor microenvironment (TME) [6]. PD-L1 immunohistochemistry (IHC) has been approved by the Food and Drug Administration (FDA) as a biomarker for treatment with anti-PD-1 therapies [7]. Nevertheless, the majority of patients derives limited benefit from immunotherapy despite high PD-L1 expression, while those with low PD-L1 expression still respond [7, 8]. The predictive value of tissue PD-L1 expression remains controversial possibly due to tumor heterogeneity. Discordance between the primary and metastatic lesions, in cooperation with the discrepancy of various metastatic sites and intra-organ lesions further implies that tissue PD-L1 expression is not a perfect biomarker [9–13]. Moreover, even sample types (e.g., biopsy versus resection) contribute to the observed differences [14]. Moreover, pMMR CRC patients undergoing immunotherapy generally experience heavy treatments. Radiotherapy and chemotherapy reportedly may influence tissue PD-L1 expression [15–17], but the inaccessibility of samples prevents the re-evaluation of PD-L1 expression after treatment. Thus, new biomarkers are urgently needed to predict efficacy.

Recently, soluble forms of PD-L1 (sPD-L1), which include exosomal PD-L1 (exo-PD-L1) and secreted splice variants (secPD-L1), have been identified in the peripheral blood and proven to inhibit the functions of T cells, meditate tumor evasion, and promote tumor progression [18]. sPD-L1 is easily detected in the blood with noninvasive measurements. sPD-L1 has been proposed as a prognostic marker to predict recurrence and survival in various tumors [19–22]. Importantly, sPD-L1 and exoPD-L1 have been recognized as biomarkers to predict the efficacy of immunotherapy in melanoma [23–27], non-small cell lung cancer (NSCLC) [28] and renal cell carcinoma (RCC) [25].

Limited studies have evaluated the role of sPD-L1 in CRC. Recently, three alternative splicing isoforms of secPD-L1 have been identified and their functions were assessed in a preclinical model of CRC [29]. In contrast to the other two forms, PD-L1 isoform a mainly regulates colorectal cancer stem cell (CSC) expansion. PD-L1 isoform b significantly inhibits T-cell function and meditates tumor evasion. Isoform c promotes tumor proliferation, migration and invasion through epithelial-mesenchymal transition (EMT). Moreover, it can also bind to PD-1 and inhibit T-cell activity, although to a lesser extent than isoform b. Higher serum secPD-L1 level has been further verified to indicate poor prognosis in CRC patients [29]. An elevated sPD-L1 level has been found in patients with CRC compared with healthy controls, and in CRC patients with local lymph node metastasis compared with those without local lymph node metastasis [30]. In another study that assessed the prognostic effects of serum PD-L1 and cytotoxic T-lymphocyte antigen 4 (CTLA-4) in stage I–III CRC, elevated levels of serum PD-L1 indicated inferior disease-free survival (DFS) and overall survival (OS) [31]. In addition, Chen et al. revealed that both preoperative exoPD-L1 and sPD-L1 were associated with T-cell infiltration and predicted poorer prognosis in patients with colorectal liver metastasis (CRLM) after hepatic resection [32]. Moreover, serum sPD-L1 levels significantly increased after chemoradiotherapy (CRT) in patients with locally advanced rectal cancer (LARC) [33, 34]. Thus, no study has fully estimated sPD-L1 levels in metastatic CRC (mCRC), except those with resectable CRLM. Furthermore, whether systemic therapies such as chemotherapy and targeted therapy influence the sPD-L1 levels remains unknown. More importantly, the role of sPD-L1 in predicting the tumor response to immunotherapy in patients with CRC has not been evaluated.

In this study, we aimed to analyze the association between sPD-L1 and clinicopathological features, including tissue PD-L1 IHC staining. We further explored the dynamic changes during systemic therapy and their predictive value for combination immunotherapy in CRC.

## Methods

### Patient selection and blood collection

**Fig. 1:**
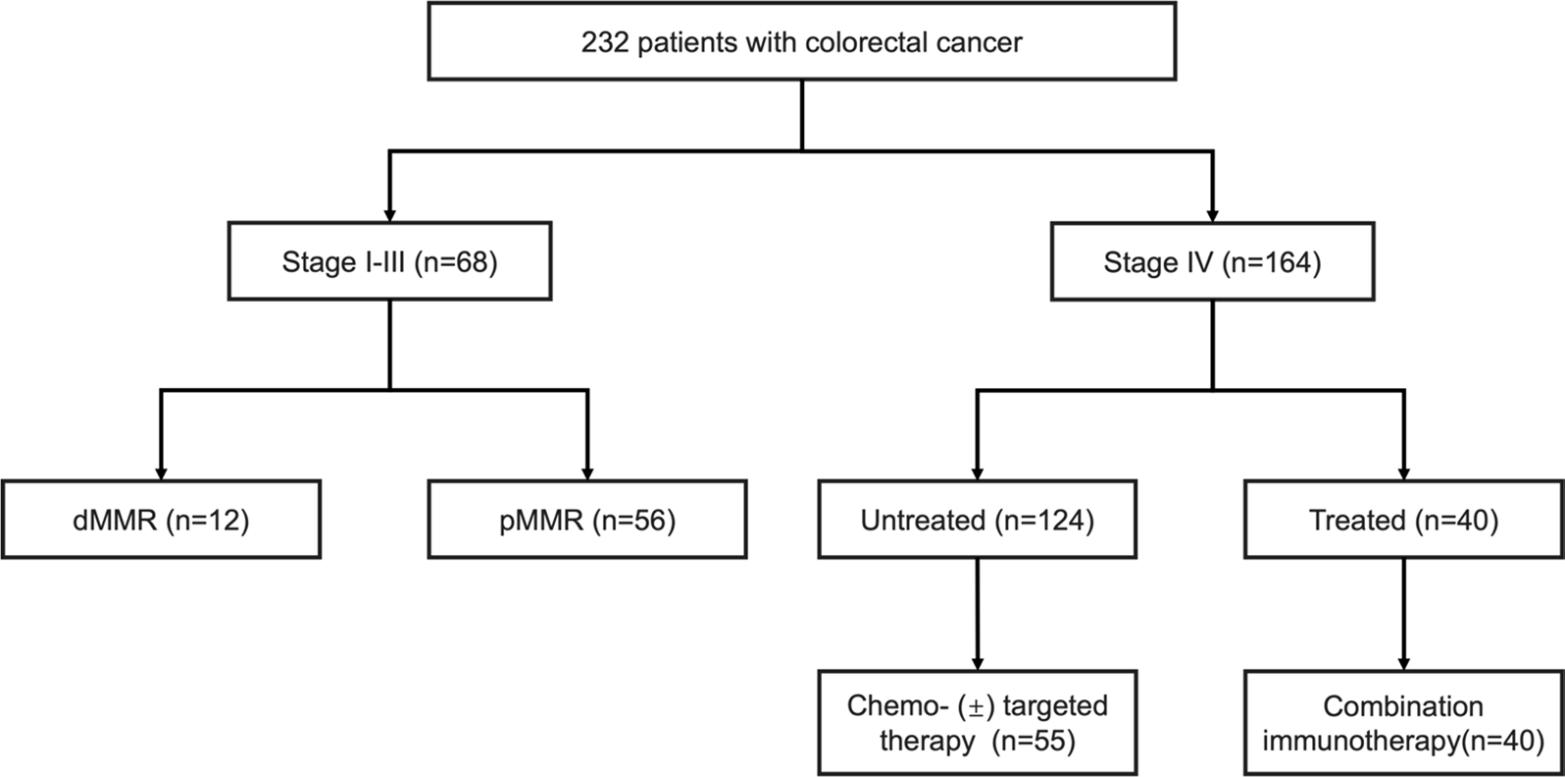
Schematic diagram for the composition of samples in our study

The study enrolled a total of 232 patients (Fig. 1) admitted to the Department of Colorectal Surgery of First Affiliated Hospital, Zhejiang University School of Medicine, China, from December 2020 to August 2022. Sixty-eight patients who were primarily diagnosed with stage I–III CRC and underwent radical surgery, together with 124 patients with pMMR metastatic colorectal cancer (mCRC), were included in our study. Another 40 patients with refractory pMMR CRC, who received regorafenib combined with sintilimab, a PD-1 inhibitor, were also enrolled in our study. To fully assess the dynamic changes in sPD-L1 levels during treatment, plasma samples from patients with mCRC who received systemic therapy were obtained at baseline and cycle 4 (cycle 8 and cycle 12 were also obtained in those with mCRC who received chemotherapy or targeted therapy). Written informed consent was obtained before enrollment. The study was approved and supervised by the Ethics Committee of the First Affiliated Hospital, Zhejiang University School of Medicine.

Peripheral blood samples were collected into EDTA tubes and isolated cell-free plasma samples were stored at – 80 ℃.

### Response evaluation

The response was assessed in accordance with the Response Evaluation Criteria in Solid Tumors (RECIST) version 1.1 [35]. Durable clinical benefit (DCB) was defined as complete response (CR), partial response (PR) or stable disease (SD) that lasted more than 6 months. Progression-free survival (PFS) was defined as the time interval between the initial dose and the first recorded progression or death from any cause. Overall survival (OS) was defined as the time from enrollment to death from any cause.

### Isolation and characterization of exosomes

Thawed plasma samples (1 mL) were differentially centrifuged at 360 × *g* for 20 min at 4 ℃, 2000 × *g* for 15 min at 4 ℃ and then 12,000 × *g* for 30 min at 4 ℃. The collected supernatant was ultracentrifuged at 200,000 × *g* for 2 h at 4 ℃ (Beckman Coulter, Optima MAX-XP; MLA-130 rotor). The supernatant was collected as secPD-L1. The pellet was washed with 1 mL of PBS and followed by a second ultracentrifugation at 200,000 × *g* for 2 h at 4 ℃. The supernatant was discarded and the exosomes were resuspended in 100 µL of PBS.

The size and concentration of exosomes were detected by nanoparticle tracking analysis (NTA) using a NanoSight NS300 instrument (Malvern Instruments). The morphology of exosomes was examined using transmission electron microscopy. The isolated exosomes were placed on a copper grid and negatively stained with uranyl acetate. Images were acquired using a JEM-1400 (JEOL) at 80 kV voltage.

Exosome lysates were obtained by adding radioimmunoprecipitation assay (RIPA) buffer with protease inhibitors and the protein concentration was determined by BCA protein assay kit (Thermo Scientific). Approximately 20 μg of total exosome protein was electrophoresed on a 10% sodium dodecyl sulfate–polyacrylamide gel and electrotransferred onto a PVDF membrane (Millipore). The membrane was blocked with 5% milk for 2 h, incubated overnight at 4 ℃ with antibodies specific for PD-L1 (1:1000, ab205921, Abcam), TSG-101 (1:1000, ab125011, Abcam) and CD63 (1:1000, ab193349, Abcam), and the incubated with a horseradish peroxidase-conjugated secondary antibody. The blots on the membrane were developed with ECL detection reagents (Pierce) and captured using Chemidoc MP (Bio-Rad).

### Determination of PD-L1 concentration in plasma

sPD-L1 and secPD-L1 concentrations were measured using an enzyme-linked immunosorbent assay (PD-L1 Human ELISA Kit, Abcam) in accordance with the manufacturer’s instructions. The detection range was from 7.81 pg/mL to 500 pg/mL and measurements below the detection limit were recorded as “7.81 pg/mL”.

### PD-L1 detection on exosomes by flow cytometry

The staining method used for flow cytometry of exosomes coupled to beads was modified based on the methods described by Morales–Kastresana [36] and Theodoraki [37]. Briefly, 10 µg exosome protein was coincubated with 1 µg biotin-labeled anti-CD63 antibody (353,018, Biolegend) for 2 h at room temperature. Next, 15 µL of streptavidin-coated magnetic beads (MBL International) was added and the compounds were gently agitated on a shaker for 2 h at room temperature. The samples were washed once with dilution buffer from the kit and then coincubated with 10 µL of the detection antibody anti-PD-L1 PE (329706, Biolegend) or the labeled isotype control antibody (400314, Biolegend) for 1 h at room temperature. The complexes were resuspended in 100 µL PBS after washing them three times with dilution buffer for antigen detection (Beckman Coulter CytoFlex). The lower edge of the “positive gate” was set at the point where  < 2% of the isotype control was positive.

### Tissue PD-L1 expression quantification

Formalin-fixed paraffin-embedded (FFPE) tissue containing histologically confirmed colorectal cancer was retrieved and subjected to immunohistochemistry (IHC) staining with an anti-human PD-L1 monoclonal antibody (22C3, Dako) to assess PD-L1 expression. Tumor proportion score (TPS)  ≥ 1% and combined positive score (CPS)  ≥ 10 were considered “positive” in our study.

### Statistical analysis

Categorical variables were compared by the chi-square test or Fisher’s exact test. Continuous variables were compared using the Mann–Whitney test, the Kruskal–Wallis test or the Wilcoxon matched-pairs test. Survival data were estimated by the Kaplan–Meier method and tested the difference was tested by the log-rank test. Correlations were determined by the Spearman coefficient. Cutoff values for continuous variables were determined based on the Receiving Operating Curve (ROC) method. A two-tailed p value  < 0.05 was considered statistically significant. Statistical analyses were performed using SPSS statistical software (version 26; IBM) and GraphPad Prism 9 software.

## Results

### SecPD-L1, rather than exoPD-L1 is the major component and positively correlated with sPD-L1 in CRC

**Fig. 2:**
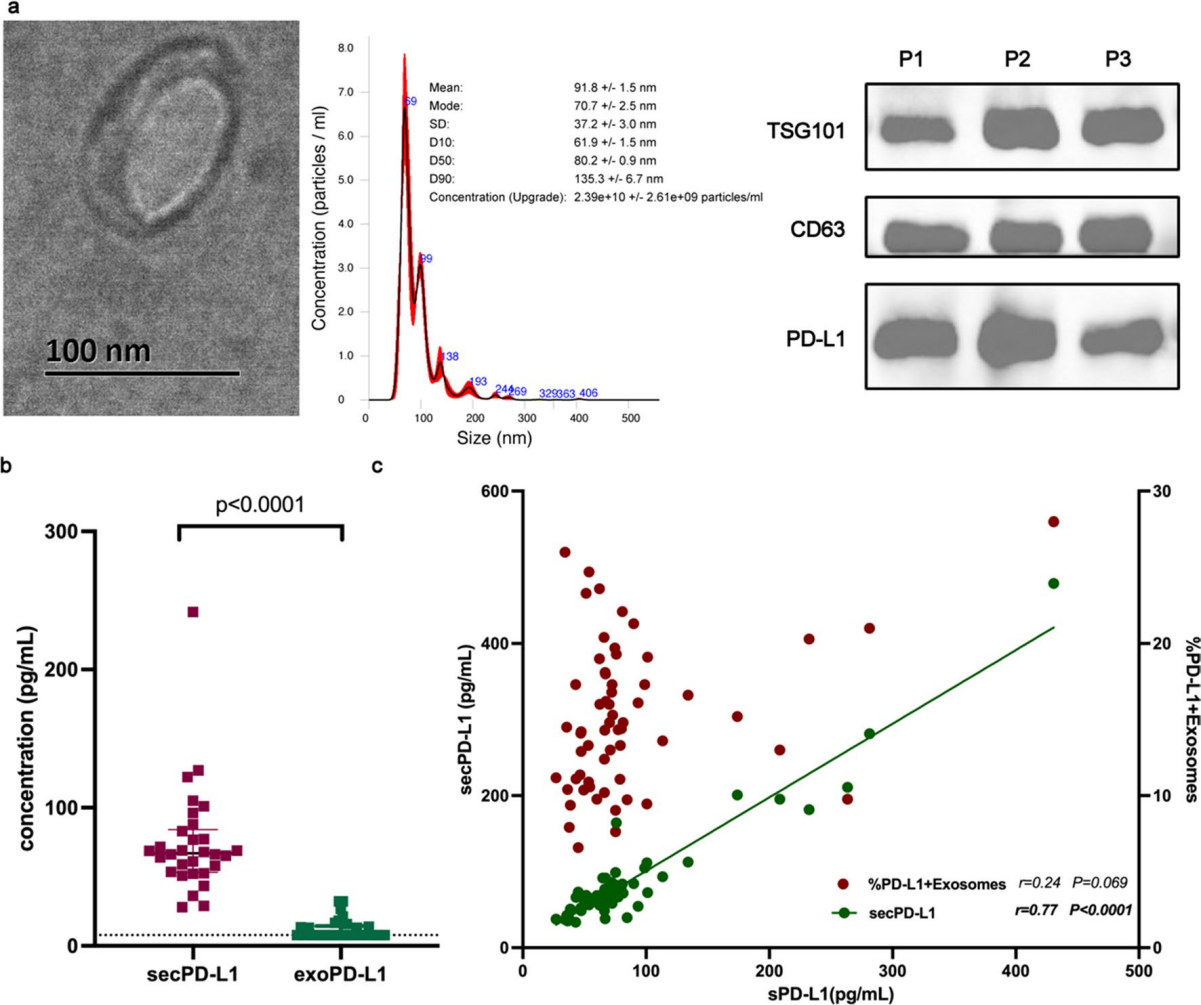
Different forms of soluble PD-L1 in patients with stage I-III colorectal cancer. **a** PD-L1 expression in exosomes characterized by transmission electron microscope (TEM), nanoparticle tracking analysis (NTA) and western blot (WB). **b** secPD-L1 level was significantly higher than exoPD-L1 (n = 30) and secPD-L1 was positively correlated to sPD-L1 (n = 60). *R* and *P* were calculated by Spearman correlation

We first isolated secPD-L1 and exoPD-L1 (Fig. 2a) from 30 patients and measured their levels by ELISA. The level of secPD-L1 (median 67.09 pg/mL) was significantly higher than that of exoPD-L1 (median 7.81 pg/mL), which was barely detectable and below the detection range even in 19 patients (Fig. 2b). Considering the limitation of the detection range of ELISA, we further utilized flow cytometry to quantify the level of exoPD-L1.

To assess the levels of different forms of plasma sPD-L1 in CRC, we first enrolled 68 patients with stage I–III CRC. The levels of sPD-L1 and secPD-L1 were measured by ELISA. The secPD-L1 concentration significantly correlated with the sPD-L1 concentration, while no correlation was found between the levels of exoPD-L1 and sPD-L1 (Fig. 2c). Correlations between sPD-L1, secPD-L1 and exoPD-L1 concentrations and clinicopathological features were also investigated (Table 1). While no significant correlation was found in the sPD-L1 cohort (the plasma samples of six patients were inadequate, and only the levels of secPD-L1 and exoPD-L1 were detected), secPD-L1 was positively associated with mucinous adenocarcinoma or signet-ring cell carcinoma. The exoPD-L1 level tended to increase in patients with dMMR status (p = 0.056), which might be constricted by the small sample size with only 12 dMMR patients enrolled. Noticing numerous increases in all plasma PD-L1 concentrations in dMMR tumors, we further conducted a correlation analysis only in the pMMR cohort (n = 56, Additional file 1: Table S1). No significant correlation was observed between the sPD-L1, secPD-L1 or exoPD-L1 concentrations and clinicopathological features in the pMMR cohort, including the association between secPD-L1 and histological features. Thus, secPD-L1 functioned as the major component and positively correlated with sPD-L1, while exoPD-L1 did not correlate with sPD-L1 and might play a special role in dMMR tumors. With no conspicuous feature of different forms of plasma PD-L1 in pMMR CRC, we only analyzed the role of sPD-L1 in the subsequent analysis.

**Table 1:** Correlations between different forms of soluble PD-L1 and clinicopathological features in patients with stage I-III colorectal cancer

	sPD-L1			secPD-L1			exoPD-L1		
	High ***N*** (%)	Low ***N*** (%)	*p*-value	High ***N*** (%)	Low ***N*** (%)	*p*-value	High ***N*** (%)	Low ***N*** (%)	*p*-value
Gender						0.324			0.324
Male	20 (55.56)	20 (55.56)		22 (55.00)	18 (45.00)		22 (55.00)	18 (45.00)	
Female	9 (20.00)	9 (20.00)	0.427	12 (42.86)	16 (57.14)		12 (42.86)	16 (57.14)	
Age						***0.089***			0.808
≤ 60	12 (42.86)	12 (42.86)		13 (39.39)	20 (60.61)		16 (48.48)	17 (51.52)	
> 60	17 (53.13)	17 (53.13)	1.000	21 (60.00)	14 (40.00)		18 (51.43)	17 (48.57)	
Location of primary						0.253			0.253
Left	24 (48.00)	24 (48.00)		28 (53.85)	24 (46.15)		28 (53.85)	24 (46.15)	
Right	5 (50.00)	5 (50.00)	***0.073***	6 (37.50)	10 (62.50)		6 (37.50)	10 (62.50)	
Histology						**0.021**			0.323
Adenocarcinoma	21 (42.86)	21 (42.86)		25 (43.86)	32 (56.14)		27 (47.37)	30 (52.63)	
MC or SRCC	8 (72.73)	8 (72.73)	0.915	9 (81.82)	2 (18.18)		7 (63.64)	4 (36.36)	
Differentiation						0.720			0.162
Poor	6 (46.15)	6 (46.15)		8 (53.33)	7 (46.67)		5 (33.33)	10 (66.67)	
Well/Moderate	22 (47.83)	22 (47.83)	0.796	25 (48.08)	27 (51.92)		28 (53.85)	24 (46.15)	
Tumor size (cm)						0.627			0.627
≧4.5	15 (50.00)	15 (50.00)		17 (51.13)	15 (46.88)		15 (46.88)	17 (53.13)	
< 4.5	14 (46.67)	14 (46.67)	0.511	17 (47.22)	19 (52.78)		19 (52.78)	17 (47.22)	
T						0.300			1.000
T1-2	8 (42.11)	8 (42.11)		9 (40.91)	13 (59.09)		11 (50.00)	11 (50.00)	
T3-4	21 (51.22)	21 (51.22)	0.897	25 (54.35)	21 (45.65)		23 (50.00)	23 (50.00)	
Lymph node metastasis						1.000			0.549
N0	23 (47.92)	23 (47.92)		27 (50.00)	27 (50.00)		28 (51.85)	26 (48.15)	
N1-2	6 (50.00)	6 (50.00)	1.000	7 (50.00)	7 (50.00)		6 (42.86)	8 (57.14)	
Perineural invasion						0.692			0.771
Absent	24 (48.98)	24 (48.98)		26 (47.27)	29 (52.73)		27 (49.09)	28 (50.91)	
Present	5 (55.56)	5 (55.56)	0.203	6 (60.00)	4 (40.00)		6 (60.00)	4 (40.00)	
Vessel invasion						0.200			0.871
Absent	17 (43.59)	17 (43.59)		19 (43.18)	25 (56.82)		21 (47.73)	23 (52.27)	
Present	10 (62.50)	10 (62.50)	0.897	11 (61.11)	7 (38.89)		9 (50.00)	9 (50.00)	0.549
TNM classification						1.000			
Stage I/II	23 (47.92)	23 (47.92)		27 (50.00)	27 (50.00)		28 (51.85)	26 (48.15)	
Stage III	6 (50.00)	6 (50.00)	0.438	7 (50.00)	7 (50.00)		6 (42.86)	8 (57.14)	***0.056***
MMR status						0.203			
dMMR	7 (58.33)	7 (58.33)		8 (66.67)	4 (33.33)		9 (75.00)	3 (25.00)	0.324
pMMR	22 (45.83)	22 (45.83)		26 (46.43)	30 (53.57)	0.324	25 (44.64)	31 (55.36)	

Bold refers *p* < 0.05 with statistical significance

Bold italic refers *p* > 0.05 but < 0.10 with potentially statistical significance

*MC* mucinous adenocarcinoma,* SRCC* signet-ring cell carcinoma,* dMMR* deficient mismatch repair,* pMMR* proficient mismatch repair

### sPD-L1 positively correlates with distant metastasis, especially distant lymph node metastasis and tissue CPS

**Fig. 3:**
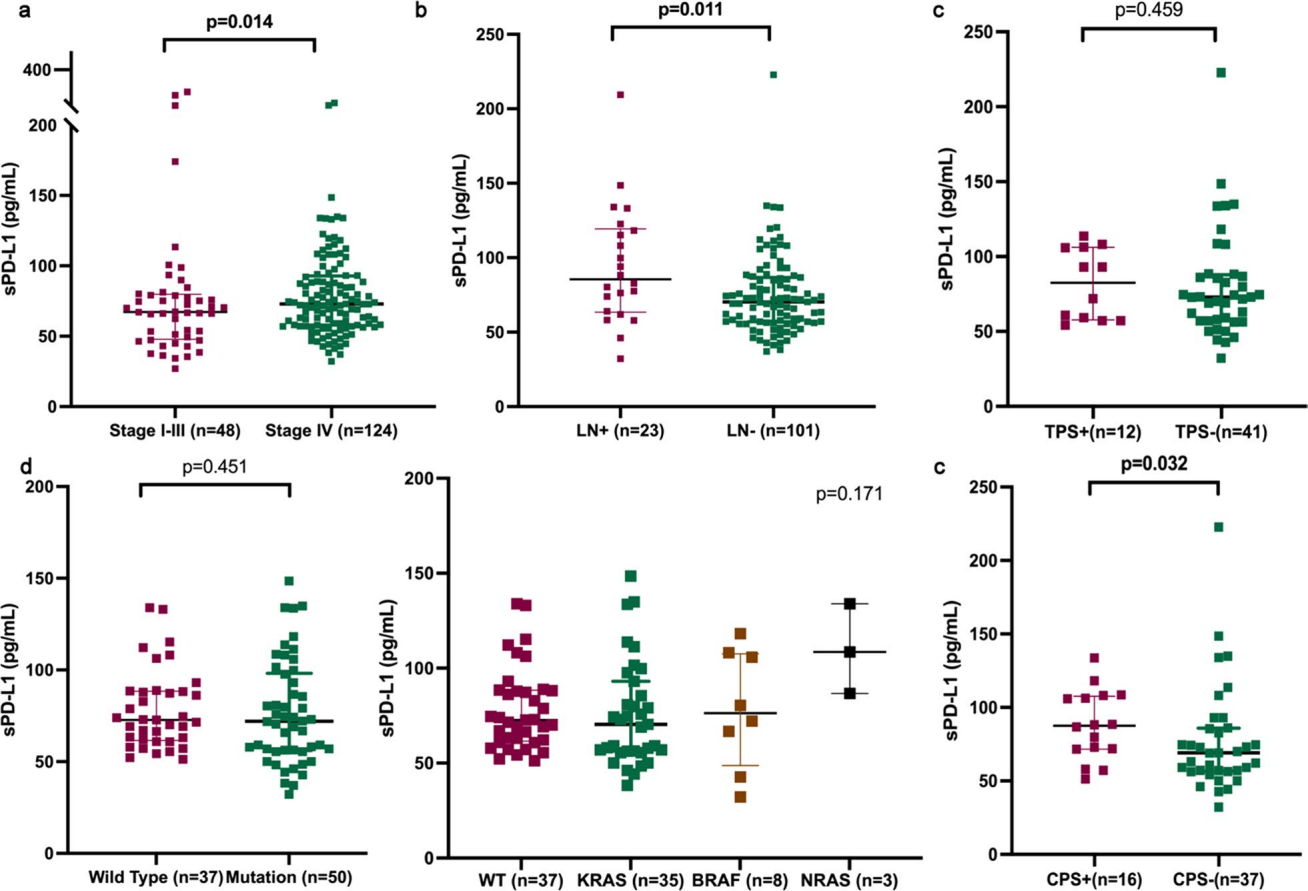
Comparisons of soluble PD-L1 in patients with metastasis colorectal cancer. **a** sPD-L1 was significantly elevated in metastatic tumors. **b** Distant lymph node metastasis indicated higher level of sPD-L1. **c** CPS positive was associated with increased sPD-L1 concentration while no correlation was found between TPS and sPD-L1. **d** No correlation was found between RAS/BRAF mutation and sPD-L1 concentration

To evaluate the role of sPD-L1 in metastatic pMMR CRC, we enrolled 124 patients and investigated the correlation between sPD-L1 concentration and clinical features mainly metastatic sites (Table 2). The level of sPD-L1 significantly increased in metastatic patients compared with stage I–III patients (Fig. 3a). Among the different metastatic sites, only distant lymph node metastasis was positively associated with the sPD-L1 concentration (Table 2; Fig. 3b). With 56 patients available for tissue PD-L1 expression detection, CPS positivity was associated with increased sPD-L1 concentration while no correlation was found between TPS and sPD-L1 (Fig. 3c). We estimated whether prevalent genomic mutations correlated with sPD-L1 concentration. No correlations were found between RAS/BRAF mutation (Fig. 3d), the top 20 common mutations in CRC (Additional file 1: Figure S1, data not shown) and sPD-L1 concentration.

**Table 2:** Correlations between sPD-L1 and clinicopathological features in patients with metastatic colorectal cancer

	***N***	High ***N*** (%)	Low ***N*** (%)	*p*-value
Gender				0.735
Male	82	28 (46.34)	44 (53.66)	
Female	42	23 (54.76)	19 (45.24)	
Age				0.879
≤ 60	52	26 (50.00)	26 (50.0)	
> 60	72	35 (48.61)	37 (51.39)	
Metastasis				0.347
Synchronous	111	53 (47.75)	58 (52.25)	
Metachronous	13	8 (61.54)	5 (38.46)	
Location of primary				0.187
Right	34	20 (58.82)	14 (41.18)	
Left	90	41 (45.56)	49 (54.44)	
Numbers of metastatic sites				***0.080***
1	92	41 (44.57)	51 (55.43)	
≥ 2	32	20 (62.50)	12 (37.50)	
Liver metastasis				0.809
Yes	80	40 (50.00)	40 (50.00)	
No	44	21 (47.73)	23 (52.27)	
Lung metastasis				0.809
Yes	29	13 (44.83)	16 (55.17)	
No	95	48 (50.53)	47 (49.47)	
Peritoneum metastasis				0.157
Yes	26	16 (61.54)	10 (38.46)	
No	98	45 (45.92)	53 (54.08)	
Lymph node metastasis				**0.030**
Yes	23	16 (69.57)	7 (30.43)	
No	101	45 (44.55)	56 (55.45)	

Bold refers *p* < 0.05 with statistical significance

Bold italic refers *p* > 0.05 but < 0.10 with potentially statistical significance

### Chemotherapy or targeted therapy has no effect on the level of sPD-L1

**Fig. 4:**
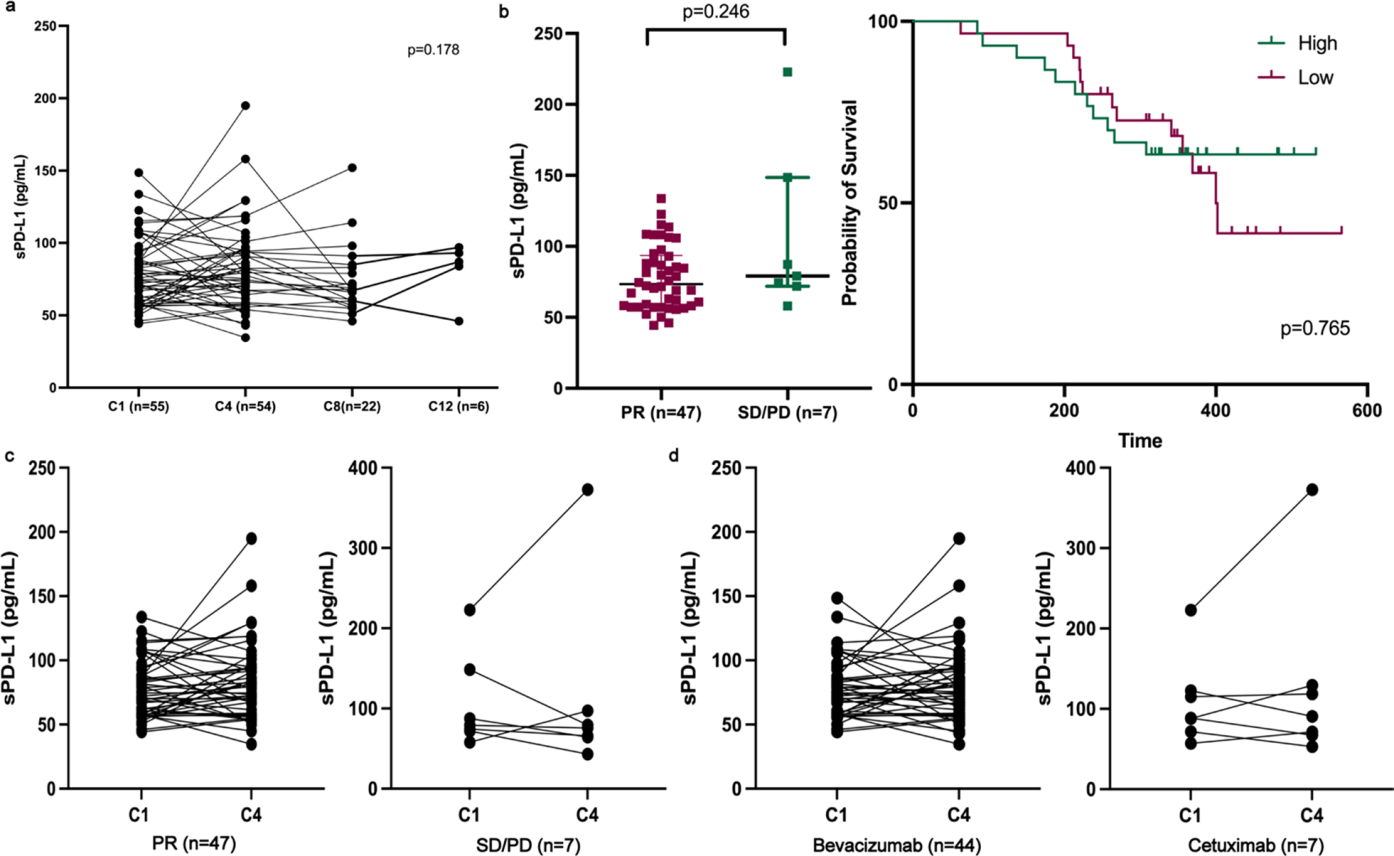
Dynamic change of soluble PD-L1 level after chemotherapy or targeted therapy. **a** No significant change was observed after systemic therapies. **b** sPD-L1 level showed no difference grouped by different response to chemotherapy or targeted therapy. Dynamic change of sPD-L1 level from cycle 1 (C1) to cycle 4 (C4) showed no significant change in different response subgroups **c** and targeted drug subgroups **d**

Among 124 patients with mCRC, 55 patients received first-line treatment at our hospital with response assessment available. Patients were treated with FOLFOXIRI + bevacizumab (n = 28), mFOLFOX6 + bevacizumab (n = 14), mFOLFOX6 + cetuximab (n = 7), FOLFIRI + bevacizumab (n = 2) and mFOLFOX6 (n = 4). No significant dynamic changes were observed after systemic therapies (Fig. 4a). Additionally, baseline sPD-L1 and dynamic changes from cycle 1 (C1) to cycle 4 (C4) failed to discriminate the efficacy of systemic therapies (Fig. 4b, c). Targeted drugs including bevacizumab and cetuximab had no effect on sPD-L1 concentration (Fig. 4d).

### sPD-L1 serves as a dynamic marker of progressive disease on combination immunotherapy in pMMR CRC

**Fig. 5:**
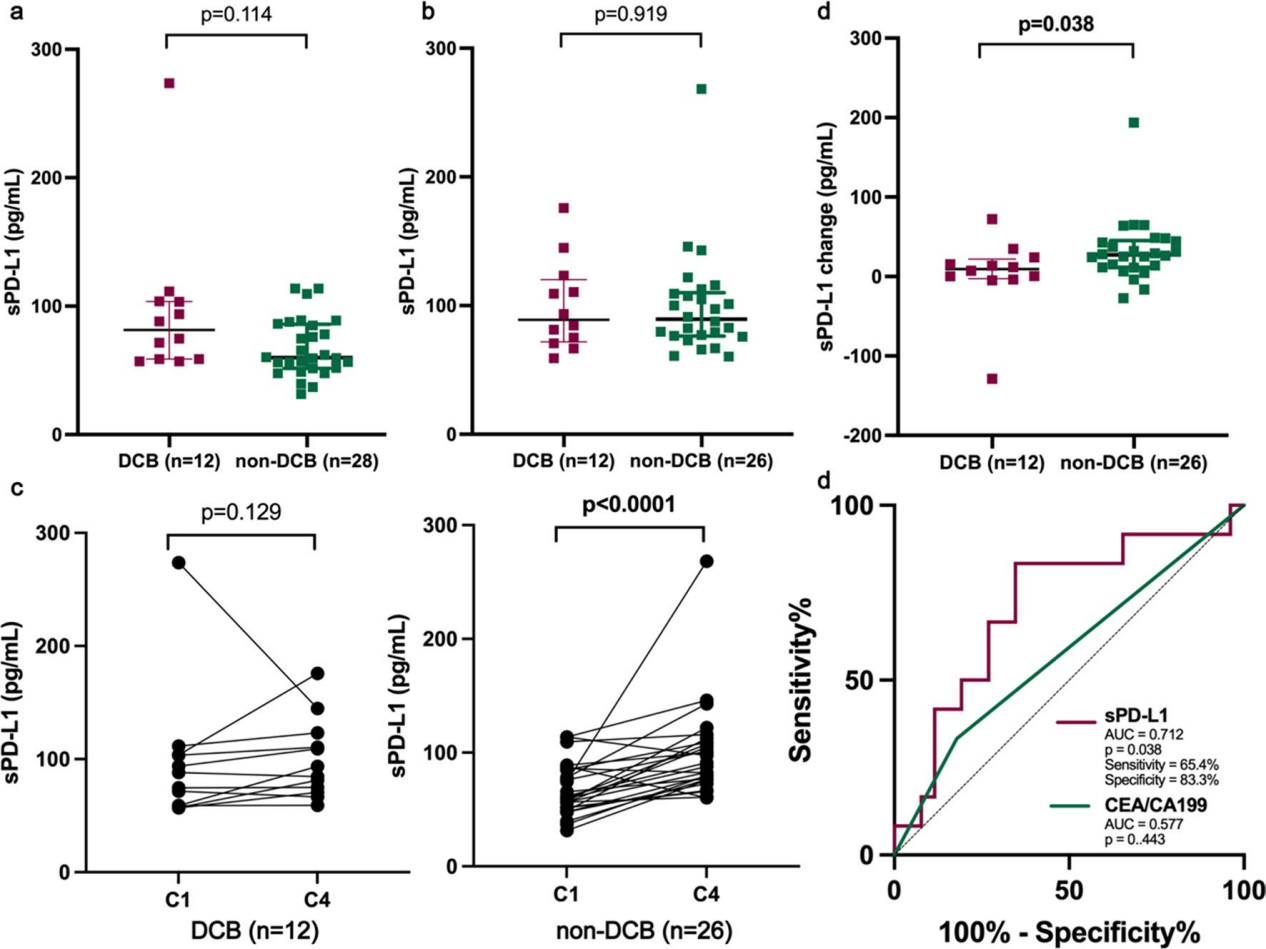
Increased level of sPD-L1 was potential to predict progressive disease on combination immunotherapy for patients with pMMR CRC. sPD-L1 level at C1 **a** or C4 **b** showed no significant difference according to treatment outcome. **c** Comparison of changes in in level of sPD-1 between C1 and C4. **d** An increase in sPD-L1 level is associated with inferior response to combination immunotherapy. C1, cycle 1; C4, cycle 4

To further explore sPD-L1 as a potential biomarker to predict the outcome of immunotherapy in pMMR CRC, we included 40 patients treated with regorafenib combined with sintilimab, a PD-1 inhibitor (two plasma samples at C4 were not acquired). The baseline clinical characteristics are displayed in Additional file 1: Table S2. Twelve patients (30%) achieved DCB. The sPD-L1 concentration at baseline (Fig. 5a) and C4 (Fig. 5b) showed no difference between the DCB and non-DCB groups. The dynamic change from baseline to C4 (∆sPD-L1) increased significantly in patients experiencing progression (n = 26), while no significant change was observed in those who achieved DCB (Fig. 5c). Furthermore, ∆sPD-L1 was significantly higher in the non-DCB cohort than in the DCB cohort (Fig. 5d). Using ROC curve analysis, ∆sPD-L1 showed good discrimination between these two cohorts [area under the curve (AUC) = 0.712, 95% confidence interval (CI) 0.529–0.894, p = 0.038]. The optimal cutoff value of ∆sPD-L1 was 24.2 pg/mL, with 83.3% specificity and 65.4% sensitivity. In contrast, conventional plasma biomarkers including CEA and CA19-9, showed inferior predictive value (AUC = 0.577; p = 0.443) (Fig. 5d).

## Discussion

In this study, we evaluated correlations between soluble forms of PD-L1 and clinicopathological features and assessed the dynamic changes after systemic treatment. To date, limited studies have assessed different soluble forms of PD-L1 in cancer, and controversial results have been reported. exoPD-L1 is more abundant than secPD-L1 in the plasma of melanoma patients [24]. A similar result has been reported in CRC, with a strong association between exoPD-L1 and secPD-L1 (r = 0.763; p < 0.001) [32]. However, comparable concentrations were observed in melanoma and gastric cancer patients [26, 38]. Moreover, exoPD-L1 was found to slightly correlated with sPD-L1 in NSCLC (r = 0.3; p = 0.0027) [39] but no correlation was found in head and neck squamous cell carcinoma (HNSCC) [37], diffuse large B-cell lymphoma [40] or extranodal NK/T-cell lymphoma [41]. In our study, we detected PD-L1 expression in exosomes; however, the concentration of exoPD-L1 was relatively low and did not correlate with sPD-L1. Conversely, secPD-L1 levels were significantly higher than exoPD-L1 levels and positively correlated with sPD-L1 levels. Exosomes isolated by size-exclusion chromatography (SEC) reportedly express more surface PD-L1 and have improved biological function compared to those obtained by ultracentrifugation (UC) [42]. Therefore, the discrepancy in the concentration of exoPD-L1 may also result from differences in the method of exosome isolation. In addition, the type of blood sample (serum versus plasma) also accounts for the difference in concentration [43]. Moreover, different detector antibodies likewise influence the detection of soluble PD-L1 [27]. According to our study, exoPD-L1 is independent of sPD-L1 and has the potential to play a role in the dMMR cohort although more studies are warranted with caution regarding isolation methods, sample preparation and detection agents.

sPD-L1 did not correlate with any clinicopathological features in our study, while a previous study revealed that only lymphatic invasion was negatively associated with sPD-L1 in stage I–III CRC patients [31]. Interestingly, we discovered an elevated sPD-L1 level in patients with distant metastasis. A previous study revealed that PD-L1 isoform c promoted metastasis by regulating EMT and weakly suppressed T-cell function [29]. Mahoney et al. reported that PD-L1 isoform c had the strongest association with full-length PD-L1 by transcriptomic analysis based on The Cancer Genome Atlas (TCGA) [44]. Thus, we hypothesize that sPD-L1, which primarily consists of PD-L1 isoform c, functions to promote tumor metastasis and regulate T-cell function in CRC. Remarkably, our study for the first time identified the positive correlation between sPD-L1 and distant lymph node metastasis. No study has compared sPD-L1 levels among different metastatic sites. With respect to tissue PD-L1 expression, metastatic lesions from lymph nodes were likely to have higher PD-L1 expression while those from bone and brain were the opposite [9–12]. The mechanism regulating different organ metastases, including lymph node metastasis, is not fully understood. A previous study suggested that only 35% of CRC cases shared common origins between lymphatic and distant metastases [45], which concurs with the findings of another study that emphasized the understanding of origins of different metastatic sites [46]. Unlike the two prevailing theories stating that lymphatic node metastasis has either a complete role or no role in the formation of distant metastases, a recent study has revealed that lymphatic node metastasis contributes to the induction of tumor tolerance and promotion of metastatic progression but is not necessary in the formation of distant metastases [47]. The upregulation of tissue PD-L1 expression has been confirmed to promote lymph node metastasis [47, 48]. Thus, we conclude that, unlike other metastatic sites, lymphatic metastases originate from unique mechanisms among which the PD-L1 pathway functions to regulate lymph node metastasis. In our study, a positive correlation with sPD-L1 level was found in distant lymph node metastasis but not in regional lymph node metastasis (RLNM). The major limitation of the study was the small sample size with only 12 patients with positive RLNM included. Additionally, considering the heterogeneous subclones in RNLM [45, 46], more studies are warranted to explore the roles of PD-L1 in regulating lymph node metastasis.

Several studies have estimated the correlation between tissue PD-L1 expression and sPD-L1 concentration and obtained controversial results. sPD-L1 levels have been revealed to not correlate with tumor PD-L1 expression in lung cancer [28, 39], brain tumors [49], pancreatic carcinoma [50], melanoma and renal cell carcinoma [25]. However, another study has found that sPD-L1 positively correlates with TPS in hepatocellular carcinoma (HCC), while no correlation has been found with PD-L1 expression on inflammatory cells [51]. In our study, we found elevated sPD-L1 concentrations in CPS-positive patients, in contrast that no significant difference was identified in the TPS cohorts. The precise origin of different forms of sPD-L1 is not yet fully understood. sPD-L1 can be secreted by numerous tumor cells and various hematologic cells, such as macrophages, activated lymphocytes and dendritic cells [52, 53]. Thus, our study further implies that sPD-L1 may represent gross PD-L1 expression in the entire tumor microenvironment including tumor and immune cells, instead of tumor cells alone. A recent meta-analysis published in *JAMA Oncology* revealed that tissue CPS, instead of TPS, is the strongest predictor of immunotherapy response in advanced gastroesophageal adenocarcinoma [54]. Interestingly, TPS has been demonstrated to be the strongest predictor in patients with squamous carcinoma [54]. A previous study also noted that PD-L1 expression on tumor-infiltrating immune cells was a better biomarker than tumor cell PD-L1 expression [55]. Thus, PD-L1 expression in immune cells, at least in some types of tumors, cooperates to regulate the tumor environment and sPD-L1 has the potential to reflect the entire inflammatory environment of the tumor.

The dynamic changes of sPD-L1 after drug exposure have rarely been estimated. Increased sPD-L1 levels were observed after radiotherapy [33, 34, 41, 56] in contrast to no significant change after anti-BRAF therapy in melanoma [23]. A previous study reported a decrease in sPD-L1 levels in patients with glioma treated by one administration of bevacizumab [57]. However, increased sPD-L1 was observed in patients with RCC who had previously been treated with vascular endothelial growth factor inhibitors (VEGFi) [25]. In our study, no significant changes were observed in patients treated with either bevacizumab, cetuximab or conventional chemotherapy. Several studies have regarded sPD-L1 as a reflection of tumor burden and investigated whether sPD-L1 predicted the efficacy of immunotherapy by simulating tumor size changes [25, 39, 56]. In our study, no correlation was observed between tumor size and sPD-L1 level. Even in the responders after chemotherapy, reduction in sPD-L1 levels was not observed. Therefore, sPD-L1 is an independent marker of tumoral inflammation features and is not associated with tumor size. Previous studies have also assessed the functions of different soluble PD-L1 forms in patients treated with checkpoint blockades, such as CTLA-4 or PD-1/PD-L1 inhibitors. Chen et al. first discovered that elevated baseline exoPD-L1 was associated with an inferior response, but an increase at weeks 3−6 indicated a better response in melanoma patients treated with pembrolizumab [26]. Elevated baseline sPD-L1 levels also indicated poor efficacy in patients with melanoma treated with CTLA-4 or PD-1 blockades [27]. However, according to another two studies, no change at the first response evaluation was observed in responders after immunotherapy, whereas a significant increase was identified in non-responders [24, 28], which is consistent with the result of our study. A recent study confirmed that a special form of secreted PD-L1, PD-L1-vInt4, acts as a decoy molecule for PD-L1 inhibitors and accounts for resistance to immunotherapy [58]. We assume that an increase in sPD-L1 levels is a reflection of a tumoral protective mechanism, which increases binding to PD-L1 inhibitors and severely impairs T-cell function. Tumors responding to immunotherapy, lack the ability to secrete adequate sPD-L1 and therefore are dampened to escape immune surveillance. Interestingly, different isoforms of secPD-L1 differentially impair T-cell function and the composition of isoforms varies among patients, which possibly contributes to the different responses to immunotherapy [29]. Only exoPD-L1 secreted from melanoma, CD8^+^ T cells and dendritic cells discriminated non-responders to checkpoint blockades while exoPD-L1 secreted from B cells and monocytes had no obvious significance in that regard [59]. Even different levels of PD-L1 expression on exosomes lead to the discrepancies in impairing T-cell function [37]. Therefore, although promising in predicting the efficacy of immunotherapy, the heterogeneity of sPD-L1 or exoPD-L1 should be taken into consideration in future research.

There are also some limitations in our study: First, previous studies have suggested that soluble PD-L1 has the potential to function as an early marker to predict the response to immunotherapy. However, we did not collect dynamic samples at cycle 2 or 3 to fully estimate the predictive role of sPD-L1. Second, limited samples were included in our analysis to estimate the composition of different forms of soluble PD-L1 and the dynamic changes after systemic therapy and immunotherapy. Only seven non-responders to chemotherapy ± targeted therapy were included, complicating the analysis of the discrimination for different responses. Third, the isolation method used in our study was ultracentrifugation, which may have accounted for the loss of PD-L1 on exosomes. Despite the aforementioned limitations, our study provided some detailed descriptions of the correlations between sPD-L1 and clinicopathological features. Our study for the first time revealed a positive correlation of sPD-L1 with distant lymph node metastasis and tissue CPS. Moreover, we identified a dynamic biomarker to predict the efficacy of combination immunotherapy in pMMR mCRCs for the first time. As a dynamic marker, sPD-L1 overcomes the limitations (failing to identify pseudoprogression and delayed response) of traditional radiological assessment in immunotherapy. sPD-L1 may help to identify rapid progression on combination immunotherapy to avoid ineffective treatment, but studies of larger cohorts are warranted to further determine whether an early increase in sPD-L1 level at C2 may identify non-responders.

## Supplementary Information

**Additional file 1.** **Figure S1.** Pretreatment of sPD-L1 level and genomic alterations from 54 patients with metastatic colorectal cancer. The plot is modified from an open-source template (https://github.com/ptgrogan/excel-oncoplot). **Table S1.** Correlations between different forms of soluble PD-L1 and clinicopathological features in patients with stage I-III pMMR colorectal cancer. **Table S2.** Baseline clinical characteristics of patients with proficient mismatch repair (pMMR) colorectal cancer treated by regorafenib combined PD-1 inhibitor.

## Data Availability

In order to protect the privacy of the patients, individual data is only available upon reasonable request in accordance to corresponding regulatory.

## Abbreviations

PD-1: Programmed cell death-1
PD-L1: Programmed cell death ligand-1
dMMR: Deficient mismatch repair
CRC: Colorectal cancer
pMMR: Proficient mismatch repair
TME: Tumor microenvironment
IHC: Immunohistochemistry
FDA: Food and Drug Administration
sPD-L1: Soluble PD-L1
exoPD-L1: Exosomal PD-L1
secPD-L1: Secreted PD-L1
NSCLC: Non-small cell lung cancer
RCC: Renal cell carcinoma
CSC: Cancer stem cell
EMT: Epithelial-mesenchymal transition
CTLA-4: Cytotoxic T-lymphocyte antigen 4
DFS: Disease-free survival
OS: Overall survival
CRLM: Colorectal liver metastasis
CRT: Chemoradiotherapy
LARC: Locally advanced rectal cancer
mCRC: Metastatic colorectal cancer
RECIST: Response evaluation criteria in solid tumors
DCB: Durable clinical benefit
CR: Complete response
PR: Partial response
SD: Stable disease
PFS: Progression-free survival
NTA: Nanoparticle tracking analysis
TPS: Tumor proportion score
CPS: Combined positive score
ROC: Receiving operating curve
TCGA: The Cancer Genome Atlas
RNLM: Regional lymph node metastasis
HCC: Hepatocellular carcinoma
VEGFi: Vascular endothelial growth factor inhibitors
