# Supplementary material for: Soluble PD-L1: a potential dynamic predictive biomarker for immunotherapy in patients with proficient mismatch repair colorectal cancer
Source: J Transl Med. 2023 Jan 13;21:25. doi: 10.1186/s12967-023-03879-0 (PMC9837921; doi:10.1186/s12967-023-03879-0)
Supplement: Supplementary file 1 — Additional file 1. Figure S1. Pretreatment of sPD-L1 level and genomic alterations from 54 patients with metastatic colorectal cancer. The plot is modified from an open-source template (https://github.com/ptgrogan/excel-oncoplot). Table S1. Correlations between different forms of soluble PD-L1 and clinicopathological features in patients with stage I-III pMMR colorectal cancer. Table S2. Baseline clinical characteristics of patients with proficient mismatch repair (pMMR) colorectal cancer treated by regorafenib combined PD-1 inhibitor. [file 12967_2023_3879_MOESM1_ESM.docx]

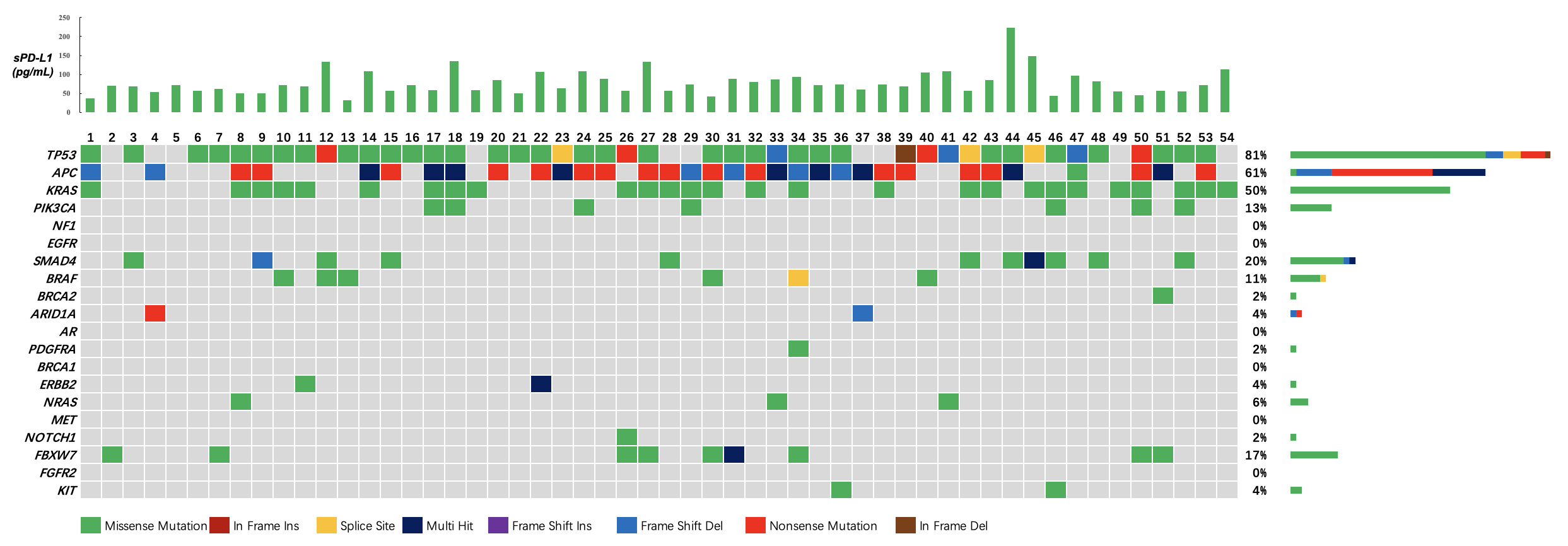


**Additional file 1: Figure S1. Pretreatment of sPD-L1 level and genomic alterations from 54 patients with metastatic colorectal cancer.**  The plot is modified from an open-source template (https://github.com/ptgrogan/excel-oncoplot).

**Additional file 1:** Table S1. Correlations between different forms of soluble PD-L1 and clinicopathological features in patients with stage I-III pMMR colorectal cancer

|  | sPD-L1 | | | | | secPD-L1 | | | exoPD-L1 | | | | |
| --- | --- | --- | --- | --- | --- | --- | --- | --- | --- | --- | --- | --- | --- |
|  | High ***N*** (%) | Low ***N*** (%) | | *p*-value | | High ***N*** (%) | Low ***N*** (%) | *p*-value | | High ***N*** (%) | Low ***N*** (%) | *p*-value |  |
| Gender |  | |  | | 0.562 | 16 (53.33)  12 (46.15)  12 (46.15)  16 (53.33)  24 (55.81)  4 (30.77)  24 (47.06)  4 (30.77)  6 (54.55)  22 (48.89)  14 (53.85)  14 (46.67)  6 (33.33)  22 (57.89)  21 (47.73)  7 (58.33)  22 (48.89)  4 (50.00)  15 (41.67)  9 (64.29)  21 (47.73)  7 (58.33) | 14 (46.67)  14 (53.85)  14 (53.85)  14 (46.67)  19 (44.19)  9 (69.23)  27 (52.94)  1 (20.00)  5 (45.45)  23 (51.11)  12 (46.15)  16 (53.33)  12 (66.67)  16 (41.11)  23 (52.27)  5 (41.67)  23 (51.11)  4 (50.00)  21 (58.33)  5 (35.71)  23 (52.27)  5 (41.67) | 0.592  0.592  0.114  0.352  0.737  0.592  0.737  1.000  1.000  0.710  0.515 | | 16 (53.33)  12 (46.15)  13 (50.00)  15 (50.00)  23 (53.49)  5 (38.46)  26 (50.89)  2 (40.00)  5 (45.45)  23 (51.11)  14 (53.85)  14 (46.67)  7 (38.89)  21 (55.26)  23 (52.27)  5 (41.67)  23 (51.11)  4 (50.00)  17 (47.22)  6 (42.86)  23 (52.27)  5 (41.67) | 14 (46.67)  14 (53.85)  13 (50.00)  15 (50.00)  20 (46.51)  8 (61.54)  25 (49.02)  3 (60.00)  6 (54.55)  22 (48.89)  12 (46.15)  16 (53.33)  11 (61.11)  17 (44.74)  21 (47.73)  7 (58.33)  22 (48.89)  4 (50.00)  19 (52.78)  8 (57.14)  21 (47.73)  7 (58.33) | 0.592  1.000  0.342  1.000  0.737  0.592  0.252  0.515  1.000  0.781  0.515 |  |
| Male  Female  Age  ≤60  > 60  Location of primary  Left  Right  Histology  Adenocarcinoma  MC or SRCC  Differentiation  Poor  Well/Moderate  Tumor size (cm)  ≧4.5  < 4.5  T  T1-2  T3-4  Lymph node metastasis  N0  N1-2  Perineural invasion  Absent  Present  Vessel invasion  Absent  Present  TNM classification  Stage I/II  Stage III | 14 (53.85)  10 (45.45)  12 (57.14)  12 (44.44)  21 (51.22)  3 (42.86)  21 (48.84)  3 (60.00)  5 (55.56)  19 (48.72)  13 (54.17)  11 (45.83)  6 (40.00)  18 (54.55)  18 (47.37)  6 (60.00)  3 (42.86)  21 (53.85)  14 (45.16)  8 (66.67)  18 (47.37)  6 (60.00) | | 12 (46.15)  12 (54.55)  9 (42.86)  15 (55.56)  20 (48.78)  4 (57.14)  22 (51.16)  2 (40.00)  4 (44.44)  20 (51.28)  11 (45.83)  13 (54,17)  9 (60.00)  15 (45.45)  20 (52.63)  4 (40.00)  4 (57.14)  18 (46.15)  17 (54.84)  4 (33.33)  20 (52.63)  3 (40.00) | | 0.383  1.000  1.000  1.000  0.564  0.350  0.477  0.694  0.206  0.477 |  |  |  |  |  |  |  |  |

MC, mucinous adenocarcinoma; SRCC, signet-ring cell carcinoma

**Additional file 1:** Table S2. Baseline clinical characteristics of patients with proficient mismatch repair (pMMR) colorectal cancer treated by regorafenib combined PD-1 inhibitor.

| Characteristics | Total, n (%) |
| --- | --- |
| Patients, N (%) | 40 (100) |
| Median age (range) | 62.5 (36-86) |
| Sex |  |
| Male | 32 (80.0) |
| Female | 8 (20.0) |
| Location of primary |  |
| Right  Left  Numbers of metastatic sites  1  >1  Metastatic sites  Liver  Lung  Peritoneum  Distant lymph node  Numbers of prior treatment lines  ≤2  >2  Genomic status  RAS/BRAF V600E wild type  RAS mutant  BRAF V600E mutant  Unknown | 7 (17.5)  33 (82.5)  16 (40.0)  24 (60.0)  20 (50.0)  20 (50.0)  18 (45.0)  11 (27.5)  26 (65.0)  14 (35.0)  12 (30.0)  22 (55.0)  2 (5.0)  4 (10.0) |
